# A case of ovarian adenosquamous carcinoma arising from endometrioid adenocarcinoma: a case report and systematic review

**DOI:** 10.1186/s13048-016-0255-6

**Published:** 2016-08-11

**Authors:** Tadahiro Shoji, Eriko Takatori, Kazuyuki Murakami, Yoshitaka Kaido, Satoshi Takeuchi, Akihiko Kikuchi, Toru Sugiyama

**Affiliations:** Department of Obstetrics and Gynecology, Iwate Medical University School of Medicine, 19-1 Uchimaru, Morioka, Iwate 020-8505 Japan

**Keywords:** Ovarian cancer, Adenosquamous carcinoma, Endometrioid adenocarcinoma, Squamous mataplasia

## Abstract

**Background:**

The aims of this report were to describe a case of ovarian adenosquamous carcinoma and to systematically review the pertinent literature.

**Methods:**

We describe a case in which a 57-year-old woman had stage IC ovarian cancer histologically diagnosed as adenosquamous carcinoma. We also systematically reviewed the literature using the PubMed database.

**Case Presentation:**

Preoperative computed tomography and magnetic resonance imaging showed a tumor measuring 14 cm in diameter and containing solid areas. Tumor marker levels were as follows: CA125, 42.6 U/mL; CA 19–9, 134.1 U/mL; CEA, 0.9 ng/mL; and SCC, 1.6 ng/mL. The patient underwent multiple surgeries including total abdominal hysterectomy, bilateral salpingo-oophorectomy, pelvic lymph node dissection, para-aortic lymph node biopsy, and total omentectomy. Based on the cytological features of the ascitic fluid, the tumor was diagnosed as a squamous cell carcinoma. Histological examination of an excised specimen showed the transition of an endometrioid adenocarcinoma to a squamous cell carcinoma. There was no evidence of any teratomas or endometriosis-related features. We considered the tumor to be an adenosquamous carcinoma, with the squamous cell carcinoma component arising from the endometrioid adenocarcinoma component. After surgery, the patient underwent 6 cycles of paclitaxel and carboplatin chemotherapy. There has been no recurrence to date, 66 months after the initial treatment.

**Results:**

Histologically, the 8 adenosquamous carcinomas reported in the literature either arose from the mature cystic teratoma (4 cases) or endometriosis (3 cases) or were pure adenosquamous carcinomas (1 case). Our literature search uncovered no cases of ovarian adenosquamous carcinomas originating from endometrioid adenocarcinomas.

**Conclusions:**

This is the first reported case of an adenosquamous carcinoma arising from an endometrioid adenocarcinoma. Because such tumors are rare, their standard management is unclear.

## Background

Various types of primary and metastatic tumors occur in the ovaries. Squamous cell carcinoma of the ovary is, however, rare, and adenosquamous carcinomas account for less than 1 % of all ovarian malignancies [1]. Conditions leading to the development of an adenosquamous carcinoma include malignant transformation of a preexisting ovarian teratoma [2, 3] and endometriosis [4]. In addition, endometrioid adenocarcinomas contain squamous components, which may become malignant. Herein we report a case of adenosquamous carcinoma that did not involve a preexisting teratoma or endometriosis. Based on the histopathological findings, we believe this tumor arose from the squamous metaplasia of an endometrioid adenocarcinoma. We describe this tumor and its histology in the context of previously reported cases obtained from a systematic review of the literature [1–7].

### Methods of literature review

**Fig. 1:**
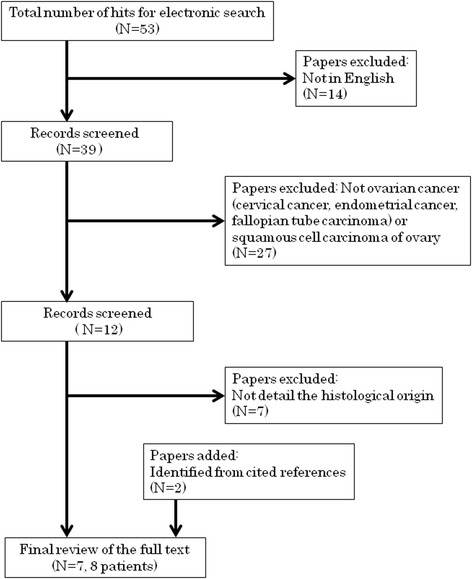
A flow chart for the systematic review in this case report

We searched the PubMed database (up to November 2015) using, among others, the key words “adenosquamous carcinoma” and “ovary.” Our search generated 53 articles; those not written in English (7 in Japanese, 3 in Chinese, and 1 each in Portuguese, Spanish, Bulgarian, and French) were excluded. Of the remaining 39 articles, 27 were excluded because the histological type was squamous cell carcinoma or endometrioid adenocarcinoma or because the cancer described was not ovarian (e.g., cervical cancer, endometrial cancer, or fallopian tube carcinoma). Of the 12 articles describing ovarian adenosquamous carcinomas, only 5 determined the histological origin of the carcinoma. The references cited by the 2 articles retrieved were also reviewed; ultimately, 7 articles reporting 8 cases are discussed herein [1–7] (Fig. 1).

## Case Presentation

We present an usual case of ovarian adenosquamous carcinoma. A primary hospital referred a 57-year-old Japanese woman (gravida 0, para 0) to our hospital because a pelvic tumor was suspected. At the primary hospital, the woman had complained of abdominal fullness.

**Fig. 2:**
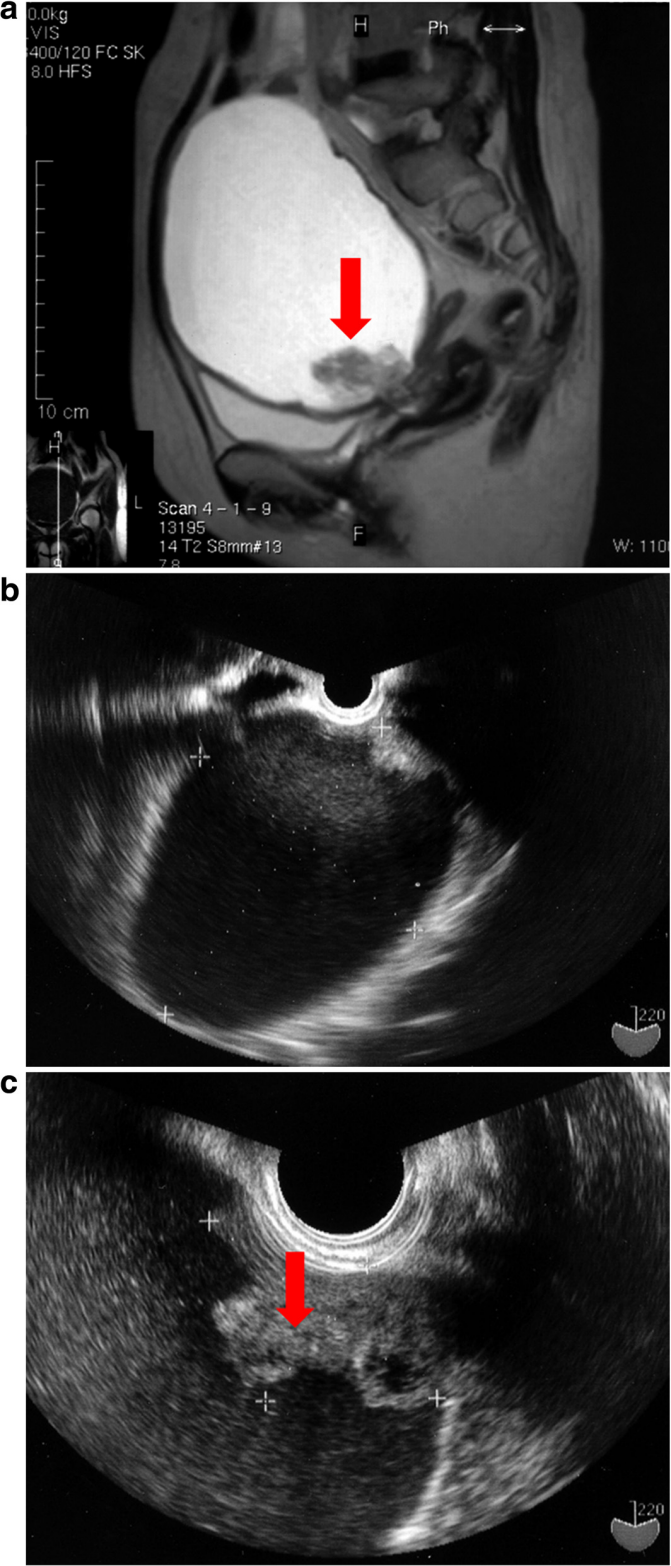
**a** Sagittal section of T2 MRI revealed a unilocular cystic tumor measuring 14 cm in diameter. There is a papillary nodule (red allow) in the tumor wall. **b** Transvaginal ultrasound revealed a unilocular cystic tumor measuring 14 cm in diameter. **c** Transvaginal ultrasound revealed a papillary nodule measuring 4 cm in the tumor wall

A cervical pap smear was negative for both intraepithelial lesions and malignancy. Digital examination revealed a mildly tender, mobile mass occupying most of the lower abdomen. Magnetic resonance imaging (Fig. 2a) conducted at the primary hospital and transvaginal ultrasound (Fig. 2b) revealed a pelvic tumor 14 cm in diameter arising from the left adnexa. In addition, a papillary nodule 4 cm in size was detected on the tumor wall, which was suggestive of malignancy (Fig. 2c). There was no clinical evidence of ascites. The tumor marker profile was as follows: CA125, 42.6 U/mL; CA19-9, 134.1 U/mL; CEA, 0.9 ng/mL; and SCC, 1.6 ng/mL. We therefore suspected ovarian cancer and performed laparotomy.

During longitudinal laparotomy, we detected a left ovarian tumor 14 cm in size and a small amount of yellow transparent ascitic fluid in the abdominal cavity. Fluid samples were submitted for cytology, left salpingo-oophorectomy was performed, and biopsy samples were obtained and frozen. The cytology of the ascitic fluid suggested squamous cell carcinoma, while the biopsy indicated adenosquamous carcinoma. Ovarian cancer was diagnosed, and the patient underwent total abdominal hysterectomy, right salpingo-oophorectomy, pelvic lymph node dissection, para-aortic lymph node biopsy, and total omentectomy.

**Fig. 3:**
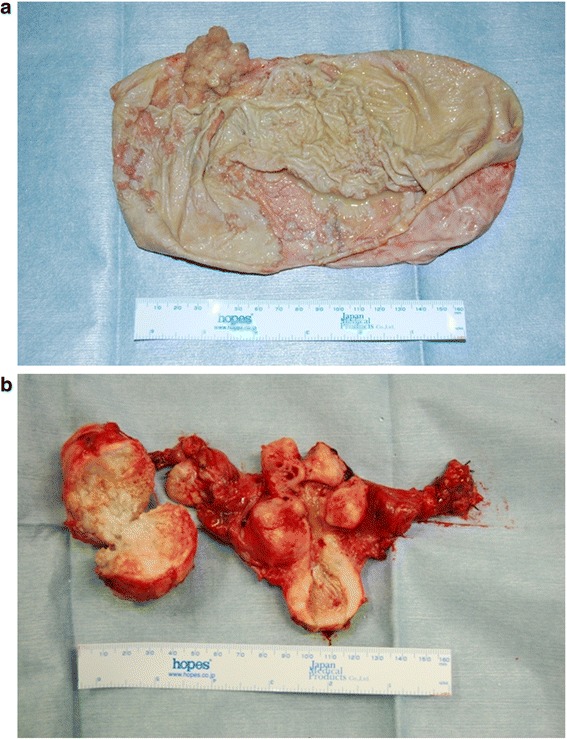
Macroscopic findings. **a** The excised ovarian tumor was unilocular and thin-walled with serous and yellow transparent fluid inside. There was a papillary nodule on the tumor wall. **b** There were numerous uterine myomas. The uterus and right adnexa showed no gross findings suggestive of malignancy

Pathological examination revealed endometrioid adenocarcinoma with squamous differentiation. Grossly, the excised specimen was unilocular and thin-walled and contained a serous, yellow transparent fluid. There was a papillary nodule in the wall of the left adnexa (Fig. 3a) and multiple uterine myomas (Fig. 3b). However, the gross findings showed no evidence of malignancy in the right adnexa (Fig. 3b), omentum, or lymph nodes. There were no concomitant teratomas or any features suggestive of endometriosis.

**Fig. 4:**
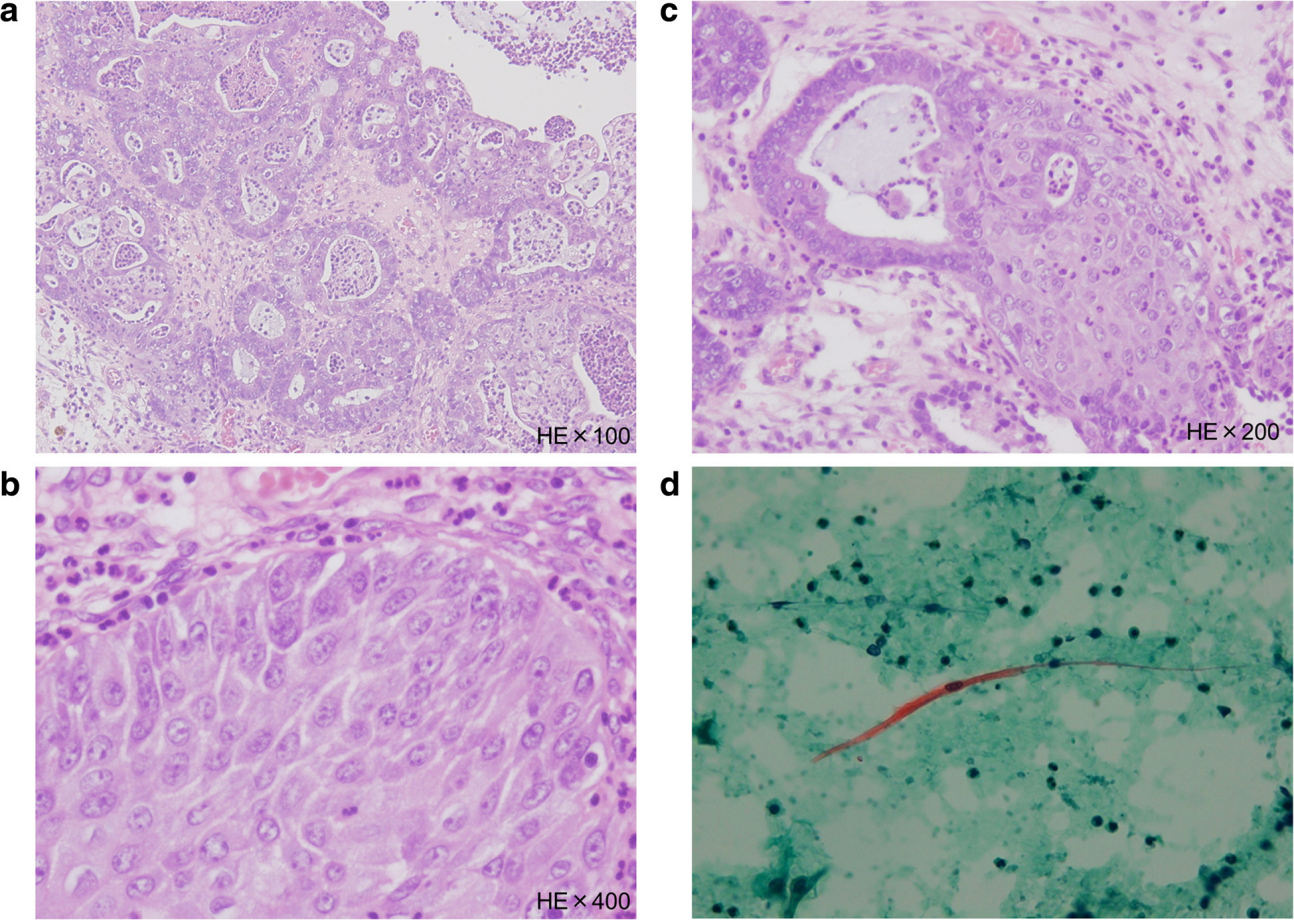
**a** Adenocarcinoma cells with glandular formation and agglutination are observed. (Hematoxylin & Eosin staining × 100). **b** Intercellular bridges are observed in cancer cells with solid growth. The tumor was diagnosed as squamous cell carcinoma. (Hematoxylin & Eosin staining × 100). **c** A transition between adenocarcinoma and squamous cell carcinoma can be seen. This strongly suggests that the squamous cell carcinoma had arisen from adenocarcinoma. (Hematoxylin & Eosin staining × 100). **d** Atypical cells are polygonal and strongly stained with orange G, indicating nuclear concentration. These findings suggest the cells to be atypical and to be derived from squamous cell carcinoma. (Papanicolaou staining × 100)

Histologically, the endometrioid adenocarcinoma (Fig. 4a) and squamous cell carcinoma (Fig. 4b) components of the tumor were intermixed, with each comprising approximately half of the tumor. Differentiation from endometrioid adenocarcinoma to squamous cell adenocarcinoma was also observed (Fig. 4c). Cytological examination of the ascitic fluid revealed malignant squamous cells (Fig. 4d) but no adenocarcinoma cells. The uterus, right adnexa, omentum, and lymph nodes had no malignant components (24 pelvic lymph nodes and 11 para-aortic lymph nodes were examined). There was no evidence of a teratoma or endometriosis in the excised tissue. The surgical stage was International Federation of Gynecology and Obstetrics (FIGO) stage Ic (2).

The postoperative recovery was unremarkable. The levels of all tumor markers were within the normal range immediately after surgery. The patient received 6 cycles of paclitaxel (175 mg/m^2^) and carboplatin (area under the curve, 6 mg/mL per min) every 3 weeks. In the five and a half years since chemotherapy, she has remained recurrence-free.

## Results

The results of the systematic literature review are shown in Table 1. The cases selected from those reviewed described the clinical features of 8 patients (age range: 32–57 years, median age: 49.5 years) with adenosquamous carcinoma (4 arising from a mature cystic teratoma, 3 developing from endometriosis, and 1 described as a pure adenosquamous carcinoma). FIGO stages were reported in 6 of the 8 cases: stage I in 4 cases, stage III in 1 case, and stage IV in 1 case. Postoperative chemotherapy was performed in 4 of the 8 cases, and all regimens were platinum-based. In the 5 patients wherein the outcome was assessed, the median overall survival time was 12 months (range: 3–36 months), and 4 of the 5 patients showed recurrence.

**Table 1:** Previously reported cases with adenosquamous carcinoma of the ovary

Author/year	Ref.	Age	Origin	Stage	Adjuvant therapy	Prognosis
Maeyama (1983)	[3]	51	Mature cystic teratoma	Ib	Carboquone + Futraful	Died 6 months
Iwaoki (1994)	[2]	57	Mature cystic teratoma	Ia	CDDP + Epi + CPA	Alive well 3 years
Hsu (1996)	[5]	49	Mature cystic teratoma	I	NR	Alive well 11 months
Karateke (2006)	[6]	40	Mature cystic teratoma	Ic	No therapy	Recurrence 12 months
Moguel (1992)	[7]	50	Endometriosis	IIIc	CDDP + 5-Fu	NR
Terada (2009)	[4]	38	Endometriosis	NR	NR	NR
Terada (2009)	[4]	53	Endometriosis	NR	NR	NR
Lee (2010)	[1]	32	PureASC	IV	PTX-CBDCA	Died 3 months
Oue case		57	Endometrioid adenocarcinoma	Ic	PTX-CBDCA	Alive well 5.5 years

*ASC* adenosquamous carcinoma, *PTX* Paclitaxel, *CBDCA* Carboplatin, *CDDP* Cisplatin, *Epi* Epirubicin, *CPA* Cyclophosphamide, *NR* Not recorded

## Discussion

Many types of malignant tumors occur in the ovaries. The most common primary malignant ovarian tumors are adenocarcinomas, whereas squamous cell carcinomas are rare and adenosquamous carcinomas are even rarer. Most reported squamous cell carcinomas presumably originated from benign cystic teratomas [8] or Brenner tumors [9]. There are few reports of pure squamous cell carcinomas of the ovary [10].

Ovarian endometriosis is a frequent cause of squamous cell carcinoma [11]. Endometrioid adenocarcinomas contain squamous elements and were previously classified as adenoacanthomas or adenosquamous carcinomas depending on whether the squamous element was benign or malignant, respectively. They have more recently been termed “endometrioid adenocarcinomas with squamous differentiation” [12].

The tumor in our case was composed of intermixed endometrioid adenocarcinoma and squamous cell carcinoma components in almost equal parts. Differentiation of the endometrioid adenocarcinoma component from the squamous cell component was also observed. We therefore believe that the endometrioid adenocarcinoma developed first, after which squamous metaplasia led to the formation of the squamous cell carcinoma. It seems reasonable to describe this case as an adenosquamous carcinoma rather than an endometrioid adenocarcinoma with squamous differentiation because nearly half of the tumor consisted of malignant squamous cells. In support, in the 76 cases recently revaluated by Lim, squamous differentiation was observed in 50 cases (76 %) [13].

Cytological examination of the ascitic fluid from patients with ovarian cancer usually reveals adenocarcinoma cells. Most previous reports of ovarian adenosquamous carcinomas did not discuss the cytology of the ascitic fluid, presumably because most cytological examinations indicate adenocarcinoma. One report, however, did note the presence of numerous clusters of adenocarcinoma cells and a few squamous cells in the ascitic fluid [14]. In contrast, in our case, the ascitic fluid contained malignant squamous cells but not adenocarcinoma cells, perhaps because squamous cell carcinoma was the predominant component of the tumor. This observation strongly suggests that the squamous cell carcinoma component arose from the adenocarcinoma component.

Although the prognosis of patients with adenosquamous carcinoma merits discussion, the low number of reported cases limits such discussion. The reported 5-year survival rate was 90 % for patients with stage I or II endometrioid adenoacanthoma, but was only 36 % for patients with stage I or II endometrioid adenocarcinoma [15]. Furthermore, of the 7 reported patients with adenosquamous carcinomas arising from benign cystic teratomas, the 3 with stage Ia disease remained alive and recurrence-free for 9 months to 5 years after surgery, while the 4 with stage Ic disease were resistant to therapy and died within 13 months [2]. Karateke described a patient with stage Ic adenosquamous carcinoma who relapsed 13 months after surgery [6]. These outcome results indicate that adenosquamous carcinoma has a very poor prognosis and that clinical stage is an important determinant of outcome.

No effective treatments for adenosquamous carcinoma have yet been established. We administered paclitaxel and carboplatin because they are the standard chemotherapeutic agents for ovarian endometrioid adenocarcinoma and squamous cell carcinoma, respectively. Since her first treatment, the patient in our case has remained alive without recurrence for 66 months. Despite the reportedly poor prognosis of patients with adenosquamous carcinomas, chemotherapy consisting of paclitaxel and carboplatin was effective in our case and merits further study.

Because ovarian adenosquamous carcinomas are extremely rare, effective chemotherapeutic regimens should be established once sufficient numbers of patients can be recruited for statistical analysis. We hope that international organizations such as the Gynecologic Oncology Group will aid in this endeavor.

## Conclusions

We presented a rare case of an ovarian adenosquamous carcinoma in which the squamous cell carcinoma component originated from the endometrioid adenocarcinoma component. Histologically, the 8 adenosquamous carcinomas reported in the literature arose from either the mature cystic teratoma (4 cases) or endometriosis (3 cases) or were pure adenosquamous carcinomas (1 case). To our knowledge, we are the first report a case of an ovarian tumor consisting of intermixed squamous cell carcinoma and endometrioid adenocarcinoma components. Because there is no established chemotherapy regimen for adenosquamous carcinoma, we administered the agents (paclitaxel and carboplatin) routinely used to treat epithelial ovarian cancers to our patient. Although ovarian adenosquamous carcinoma is rare, efforts should be made to recruit a sufficient number of patients for clinical trials aimed at identifying effective treatment protocols.
